# Is a Seismic Shift in the Landscape of PFAS Uses Occurring?

**DOI:** 10.1021/acs.est.4c01947

**Published:** 2024-04-11

**Authors:** Martin Scheringer, Ian T. Cousins, Gretta Goldenman

**Affiliations:** † Institute of Biogeochemistry and Pollutant Dynamics, ETH Zürich, 8092 Zürich, Switzerland; ‡ Department of Environmental Science, Stockholm University, SE-10691 Stockholm, Sweden; § Milieu Consulting SPRL, 1060 Brussels, Belgium

**Keywords:** per- and polyfluoroalkyl substances, fluoropolymers, fluorinated gases, nonfluorinated alternatives, trifluoroacetic acid

We argue that a seismic shift in the landscape of per- and polyfluoroalkyl substances (PFAS) uses can be observed. From conversations with representatives of the fluorochemical industry and of large brands of consumer products; from recent statements made in the general discussion among industry, consumer groups, environmental NGOs, and academic scientists; from various analyses of the availability of alternatives to PFASs in many use areas, including our own work;^1−4^ and from the decision of a major PFAS manufacturer (3M) to leave entirely the production of PFAS,^5^ we conclude that in many PFAS use areas, the transition to nonfluorinated alternatives is underway and is gaining more and more momentum.

The ongoing transition to PFAS-free alternatives includes uses such as surface treatment for a wide range of materials (food-contact materials, textiles, carpets, leather, metals, and cookware), lubrication, personal care products and cosmetics, firefighting foams, components of electrical devices (e.g., in fuel cells), ski waxes, cleaning products, building materials, refrigerants, and many other uses not highlighted here.^6^

We use the term “seismic shift” because only a few years ago the fluorochemical industry was focused on phasing out longer-chain perfluoroalkyl chemistries with the intention of shifting to the presumably long-term use of shorter-chain perfluoroalkyl and perfluoroalkylether chemistries in multiple applications.^7,8^ Uses of fluoropolymers and fluorinated gases were hardly under discussion for general chemicals regulation. Additionally, until a few years ago, the fluorochemical industry was committed to the continued production of side-chain fluorinated polymers for use in surface treatments, claiming that they were highly resistant to degradation during their lifetime and that residual low-molecular PFAS could be virtually eliminated from products.^9^

We have recently heard representatives of the fluorochemical industry state publicly at high-profile international meetings such as the FLUOROS 2023 meeting or the 2024 OECD Global Forum on PFAS that uses of low-molecular weight PFAS may be phased out and that the fluorochemical industry wants to focus on industrial uses of fluoropolymers and uses of fluorinated gases. This is supported by an industry website advocating continued use of fluoropolymers and fluorinated gases and by an attempt to exclude fluoropolymers and fluorinated gases from the definition of PFAS in the US State of Indiana.^10,11^ This focus on fluoropolymers and fluorinated gases is not surprising given that these substances are by far the two groups of PFAS with the largest production volumes among all PFAS, manufactured in millions and hundreds of thousands of metric tons, respectively.^12^ The fluoropolymer business alone was worth approximately $7.9 billion in 2023,^4^ and production and revenues are forecast to increase rapidly in the future.

For the remaining industrial uses of fluoropolymers, the fluoropolymer industry is now publicly acknowledging the need for substantial reductions in PFAS emissions during production, by adopting “responsible manufacturing” practices, and to ensure that the ultimate disposal of fluoropolymers is also carried out safely with minimal environmental emissions.^13,14^ These measures are much needed.^15,16^ However, the industry has a history of legally challenging regulatory efforts to require reductions in emissions at specific sites.^17^ It remains to be seen whether industry will voluntarily take the measures needed to reduce emissions from fluoropolymer production, recycling, and end-of-life to zero or whether regulatory pressure needs to continue.

Moreover, much is still unknown regarding the emissions of PFAS from fluoropolymer manufacturing. Emission reporting has been shown to be highly asymmetric in Europe.^16^ Large uncertainties remain with regard to emissions of polymerization byproducts, chain-transfer agents, and fluorinated solvents from fluoropolymer manufacturing. Emission reductions from fluoropolymer manufacturing should be equally stringent globally to prevent a “race to the bottom” whereby manufacturing moves to countries where regulation is less stringent.

For the fluorinated gases mentioned above, it is known that some of these are partially or completely converted into trifluoroacetic acid (TFA). The fluorochemical industry argues that TFA is not of concern^18^ as it is non-bioaccumulative and of relatively low toxicity, but they conveniently ignore the fact that (1) environmental levels of TFA have been increasing at alarming rates in recent decades,^19,20^ (2) the levels will eventually breach guidelines set for safe consumption of drinking water, and (3) TFA is practically impossible to remove from water.^21^ Moreover, it has been demonstrated that natural sources of TFA are unlikely to exist,^22^ and evidence is emerging that TFA may be a reprotoxicant.^23^ At the same time, for many uses of fluorinated gases, equivalent alternatives are on the market. We contend that a transition to using nonfluorinated refrigerants and foam blowing agents is possible and that this has happened already in many sectors.

It is important to ensure that efforts to change the broad regulatory definition of PFAS to exclude fluoropolymers and fluorinated gases from the definition^11^ are resisted. Furthermore, uses of fluoropolymers and fluorinated gases should be restricted to their essential uses as an additional effective way of reducing production and emissions of PFAS.

Although we are observing a seismic shift away from many uses of PFAS, there are still some outliers. For example, there are still findings of (high) PFAS concentrations in consumer goods such as drinking straws.^24^ We interpret these as “scattered” remaining PFAS applications that do not represent a trend. Nevertheless, it will be important to carefully monitor the occurrence and levels of polymeric and nonpolymeric PFAS in a wide range of articles and products to empirically confirm (or refute) a trend toward the decreasing use of PFAS and to ensure compliance with the new regulatory controls over PFAS uses, such as the State of Maine’s legislation requiring a phaseout of PFAS by 2030 and the PFAS restriction proposed in the EU.

The implications of this shift are wide reaching. The open and dispersive uses of many nonpolymeric PFAS are the worst possible use pattern for extremely persistent chemicals, and it is greatly important that these uses disappear, which seems now to be possible. It can be argued that the shift is happening more quickly than expected, given the contrary statements by the fluorochemical industry just several years ago. Drivers of the shift are, on the one hand, a push by regulation (and also by the multibillion dollar settlements from the thousands of lawsuits against fluorochemical manufacturers in the United States and Europe, as well as anticipated costs from further litigation^25,26^) and, on the other hand, a pull by big brands of consumer products that want to eliminate PFAS in their products.^27^ Also, the extensive communication of scientific findings on PFAS uses, health impacts and their societal costs, and the availability of alternatives by members of the scientific community has been effective because it has created a remarkable awareness among regulators, lawmakers, civil-society groups, media, and the general public.

Finally, one of the most important drivers has been the proposed PFAS restriction in the EU, which is intended to prohibit all uses of PFAS unless granted a time-limited exemption due to lack of alternatives. Importantly, the restriction proposal lists 563 alternatives to PFAS for 210 of overall 261 PFAS use areas.^1^ The 5600 comments received from industry, academics, and the public during a six-month consultation in 2023 are currently being evaluated by EU authorities. To successfully conclude the transition indicated by the current shift, it will be of utmost importance that the PFAS restriction be implemented as intended and without much delay.
